# *Fmr1* knockout disrupts multiple intrinsic properties via reduced HCN channel activity in mediodorsal thalamocortical neurons

**DOI:** 10.1113/EP092894

**Published:** 2025-09-04

**Authors:** Gregory J. Ordemann, Polina Lyuboslavsky, Alena Kizimenko, Audrey C. Brumback

**Affiliations:** ^1^ Department of Neurology Dell Medical School at The University of Texas at Austin Austin Texas USA; ^2^ Center for Learning and Memory at The University of Texas at Austin Austin Texas USA; ^3^ Department of Pediatrics Dell Medical School at The University of Texas at Austin Austin Texas USA

**Keywords:** fragile X syndrome, HCN channels, intrinsic properties, mediodorsal thalamus, prefrontal cortex, spike timing, thalamocortical dysrhythmia

## Abstract

The neurodevelopmental disorder fragile X syndrome (FXS) results from hypermethylation of the *FMR1* gene, which prevents production of the FMRP protein. FMRP modulates the expression and function of a variety of proteins, including voltage‐gated ion channels, such as hyperpolarization‐activated and cyclic nucleotide‐gated (HCN) channels, which are integral to rhythmic activity in thalamic structures. Thalamocortical pathology, particularly involving the mediodorsal thalamus (MD), has been implicated in neurodevelopmental disorders such as FXS. MD connectivity with the medial prefrontal cortex (mPFC) is integral to executive functions such as working memory and social behaviours that are disrupted in FXS. We used a combination of retrograde labelling and *ex vivo* brain slice whole‐cell electrophysiology in 40 wild‐type and 42 *Fmr1* knockout male mice to investigate how a lack of *Fmr1* affects intrinsic cellular properties in lateral (MD‐L) and medial (MD‐M) MD neurons that project to the mPFC (MD→mPFC neurons). In MD‐L neurons, *Fmr1* knockout decreased the HCN‐mediated membrane properties voltage sag and membrane after‐hyperpolarization. We also identified a delay in rebound spike timing in both complex bursts and low‐threshold spikes. In *Fmr1* knockout mice, reduced HCN channel activity in MD‐L→mPFC neurons impaired both the timing and the magnitude of HCN‐mediated membrane potential regulation. Changes in response timing might adversely affect rhythm propagation in *Fmr1* KO thalamocortical circuitry. MD thalamic neurons are crucial for maintaining rhythmic activity involved in cognitive and affective functions. Understanding specific mechanisms of thalamocortical circuit activity might lead to therapeutic interventions for individuals with FXS and other conditions characterized by thalamic dysrhythmia.

## Abstract (highlights)

- **What is the central question of this study?**
  How does *Fmr1* knockout affect the intrinsic electrophysiological properties of mediodorsal thalamic neurons that project to the medial prefrontal cortex?
- **What is the main finding and its importance?**
  In *Fmr1* knockout mice, lateral mediodorsal thalamic neurons showed reduced HCN channel activity, resulting in decreased voltage sag, dampened after‐hyperpolarizations and delayed rebound spike timing. Disruptions in HCN‐mediated rhythmic properties might contribute to the cognitive and social/emotional challenges in fragile X syndrome, with potential relevance to other neurodevelopmental disorders.

## 1 INTRODUCTION

Changes in thalamocortical connectivity are implicated in autism spectrum disorders (Nair et al., 2013; Woodward et al., 2017). Fragile X syndrome (FXS) is an autism‐associated neurodevelopmental condition characterized by cognitive and social/emotional challenges that localize, in part, to the prefrontal brain network, which includes mediodorsal thalamus (MD) and prefrontal cortex (PFC). Largely through reciprocal communication with the PFC, the MD contributes to executive functioning and social motivation, including attention, working memory, behavioural flexibility, cognitive flexibility and social motivation (Alexander & Fuster, 1973; Bolkan et al., 2017; Fuster & Alexander, 1973; Hwang et al., 2020, 2022; Kloet et al., 2020; Lee et al., 2011; Ouhaz et al., 2018; Parnaudeau et al., 2013; Rikhye et al., 2018a). Studies of executive dysfunction in FXS have identified deficits in working memory in human subjects (Lanfranchi et al., 2009) and cognitive flexibility in a mouse model of FXS (D'Hooge et al., 1997; Kooy et al., 1996; Mercaldo et al., 2023). At the cellular level, loss of *Fmr1* disrupts the excitability of the PFC neurons that provide descending inputs to the thalamus during working memory (Brumback et al., 2017; Kalmbach et al., 2015). However, whether and how the physiology of the MD neurons that provide ascending inputs to the PFC are altered by a lack of *Fmr1* remains unknown.

Hyperpolarization‐activated, cyclic nucleotide‐gated (HCN) channels are a prominent feature of thalamic physiology (Kessi et al., 2022; Zobeiri et al., 2019). HCN channels allow neurons to ‘tune in’ to specific frequency bands by acting as pacemakers and amplifying signals at those resonant frequencies (Mishra & Narayanan, 2025). HCN channels have been implicated in a wide range of neurological conditions (Benarroch, 2013; Crunelli et al., 2023; Kessi et al., 2022) and might contribute to ‘thalamocortical dysrhythmia’, which is an overarching hypothesis for the pathophysiology of neuropsychiatric conditions ranging from Parkinsonism to depression (Llinás et al., 1999; Rogachov et al., 2025). Loss of *Fmr1* has cell type‐specific effects on HCN channel activity throughout the brain. In mice, loss of *Fmr1* decreases HCN activity in layer 5 of medial prefrontal cortex (mPFC), but it increases HCN activity in the CA1 region of the hippocampus (Brager et al., 2012; Brandalise et al., 2020; Kalmbach et al., 2015). To understand thalamic rhythms and design HCN‐directed therapies (Guo et al., 2024) targeting the prefrontal thalamocortical network, understanding how loss of *Fmr1* influences HCN channel activity in specific thalamic circuits is paramount.

The two major projections from the MD to the medial PFC (MD→mPFC) arise from the medial (MD‐M) and lateral (MD‐L) subnuclei (Kloet et al., 2020; Lyuboslavsky et al., 2024). Previously, we found that owing to higher HCN channel activity, MD‐L→mPFC neurons have shorter membrane time constants, lower membrane resistance, and require stronger current injections to generate action potentials in comparison to MD‐M→mPFC neurons (Lyuboslavsky et al., 2024).

The importance of the prefrontal thalamocortical network to executive and social/emotional function suggests MD involvement in cognitive and behavioural symptoms observed in patients with FXS. We hypothesized that the absence of *Fmr1* would cause changes in intrinsic and circuit function of MD→mPFC neurons attributable, at least in part, to alterations in ion channel function, and specifically changes in HCN channels. To test this hypothesis, we used a retrograde tracer to label fluorescently the MD neurons that project to prelimbic and infralimbic cortices in the mouse (MD→mPFC neurons). In *ex vivo* thalamic slices from adult mice, we used whole‐cell current‐clamp recordings to measure subthreshold and suprathreshold physiological properties of labelled MD‐M→mPFC and MD‐L→mPFC neurons. We compared *Fmr1* knockout (KO) animals with their wild‐type (WT) littermates. Loss of *Fmr1* caused MD‐L→mPFC neurons to display less HCN channel activity, which caused a mild increase in input resistance (*R*_N_). Loss of *Fmr1* did not cause major changes in action potential generation in response to direct current injections. However, MD‐L→mPFC neurons in *Fmr1* KO mice showed dampening of HCN‐dependent membrane properties associated with the rhythmic generation of bursting activity. First, in *Fmr1* KO mice, MD‐L→mPFC neurons had lower‐amplitude post‐depolarization after‐hyperpolarizations (AHPs). Second, these same neurons showed a slowing of rebound spiking following release of hyperpolarization. These differences in cellular physiology have implications for slow‐wave oscillations in thalamic neurons, which might influence prefrontal network activity in FXS, with broad implications for sleep and cognitive function.

## 2 MATERIALS AND METHODS

### 2.1 Ethical approval

All experiments were conducted in accordance with procedures established by the Institutional Animal Care and Use Committee at The University of Texas (approval number: AUP‐2022‐00304).

### 2.2 Animals

Male mice (8–12 weeks) were used owing to the significantly greater prevalence of FXS in males. *Fmr1* het females (gift of Kimberly Huber, UT Southwestern) were crossed with wild‐type C57Bl/6J males from Jackson Labs (stock #000664) to yield *Fmr1* ^+/^ *^y^* (wild‐type) and *Fmr1* KO male mice that were group housed with same sex littermates upon weaning at postnatal day 21 in open‐topped cages in reverse lighting conditions (09.00–21.00 h dark) with ad libitum access to food and water. In this manuscript, all further references to *Fmr1* KO mice refer to male mice only.

### 2.3 Fluorescent labelling of specific neuronal populations

Fluorescent labelling of wild‐type and *Fmr1* KO MD‐L (lateral subnucleus of mediodorsal thalamus)→mPFC (medial prefrontal cortex) and MD‐M (medial subnucleus of mediodorsal thalamus)→mPFC neurons with cholera toxin subunit B (CTB, 500 µg/100 µL, Molecular Probes, Thermo Fisher Scientific), was performed as previously described (Lyuboslavsky et al., 2024). Mice were anaesthetized with 2% isoflurane and mounted in a stereotactic frame. Anaesthesia was maintained with 2% isofluorane throughout the surgical procedure. Craniotomies were made according to stereotactic coordinates relative to bregma. To label MD neurons that project to ipsilateral prelimbic and infralimbic cortices (ipsilateral mPFC), we injected fluorescently labelled CTB into the ipsilateral mPFC (Nanofil Syringe and Pump UMP3, World Precision Instruments). Coordinates for injection into ipsilateral mPFC were (in millimetres relative to bregma): −1.7 anterioposterior, +0.3 mediolateral and −2.75 dorsoventral. After needle insertion, we waited 5 min before starting the injection of 450 nL at 100 nL/min into the mPFC. We waited 5 min after the end of the injection before slowly withdrawing the syringe needle. Subcutaneous carprofen (10 mg/kg) and bupivicane (0.25%) were administered intraoperatively prior to incision. Postoperative checks were performed at least every 12 h for the first 48 h and every 24 h subsequently, depending on the recovery status of the animal. Postoperative care included carprofen every 24 h for 2 days postsurgery and every 24 h as needed for animals exhibiting signs of pain, and neomycin applied to the incision site for 5–7 days postsurgery. Animals were assessed for signs of pain or distress (e.g. abnormal posture, vocalization, wincing, poor grooming, poor eating or drinking) at least every 12 h for 48 h postsurgery and every 24 h subsequently for 1 week. Forty‐one WT animals and 55 *Fmr1* KO animals underwent this surgical procedure in the course of this study.

We waited 3–6 days following retrograde tracer injections before performing experiments. At the time of the experiments, we verified visually that retrograde tracer injections were targeted appropriately and that tracer was not present in nearby structures. Experiments were performed on neurons located in MD from bregma = −1.00 mm to bregma = −1.50 mm. Neurons recorded anterior to this range were considered part of a transition area in MD and excluded from analysis (Mátyás et al., 2014). In this manuscript, WT and *Fmr1* KO refer to ‘WT MD‐L’ and ‘*Fmr1* KO MD‐L’ unless otherwise indicated.

### 2.4 Histology

Three animals were used for histological confirmation of injection location. Mice were anaesthetized with intraperitoneal injection of ketamine/xylazine (90/10 mg/kg; Acor/Dechra) and perfused transcardially with paraformaldehyde (Sigma‐Aldrich) 4% in 1× PBS. The brain was removed and left overnight in 4% paraformaldehyde in PBS. All brain tissue was sectioned in 50‐µm‐thick coronal slices with 4′,6‐diamidino‐2‐phenylindole (DAPI)‐containing mounting medium (VectaShield HardSet with DAPI, Vector laboratories), and slices containing mPFC and MD were imaged at ×5 magnification using Zeiss Axio Imager 2.

### 2.5 Acute brain slice preparation

Slices 250 µm thick (Leica VT1200) were prepared from mice 8–12 weeks old after intraperitoneal injection of ketamine/xylazine (90/10 mg/kg; Acor/Dechra). Mice were perfused with cutting solution containing (mM): 205 sucrose, 25 NaHCO_3_, 2.5 KCl, 1.25 NaH_2_PO_4_, 7 MgCl_2_, 7 dextrose, 3 sodium pyruvate, 1.3 sodium ascorbate and 0.5 CaCl_2_ bubbled with 95% O_2_–5% CO_2_. Slices were incubated in holding solution containing (mM): 125 NaCl, 25 NaHCO_3_, 2.5 KCl, 1.25 NaH_2_PO_4_, 25 dextrose, 2 CaCl_2_, 2 MgCl_2_, 1.3 sodium ascorbate and 3 socium pyruvate at 37°C ± 1°C for 30 min, then kept for ≥30 min at room temperature before recording began.

### 2.6 Intracellular recordings

Artificial cerebrospinal fluid (ACSF) contained (mM): 125 NaCl, 25 NaHCO_3_, 12.5 dextrose, 2.5 KCl, 1.25 NaH_2_PO_4_ 2 CaCl_2_ and 1 MgCl_2_. Slices were continuously perfused with ACSF in an immersion chamber (Warner Instruments) with temperature maintained at 32.5°C ± 1°C (Warner Instruments TC‐324C). We did not add synaptic blockers to the ACSF unless otherwise specified. In a subset of experiments, ZD7288 (ZD, 20 µM; Tocris catalogue no. 1000) or TTX (0.5 µM; Abcam catalogue no. ab120055) was added to the extracellular ACSF.

Somatic whole‐cell patch recordings were obtained from retrogradely labelled neurons in the medial (MD‐M) or lateral (MD‐L) subnuclei of WT and *Fmr1* KO mice, using DODT (Zen 2.5 blue addition, Zen pro) contrast microscopy and epifluorescence on an upright microscope (Zeiss Examiner D1). Patch electrodes (tip resistance = 3–6 MΩ) were filled with the following (mM): 118 potassium gluconate, 10 KCl, 10 HEPES, 4 MgATP, 1 EGTA, 0.3 Na_3_GTP and 0.3% biocytin (pH adjusted to 7.2 with KOH; 282 mosmol/L). For some cells, 16 µM Alexa 488 or Alexa 594 was added to the internal solution to visualize the dendritic arbor under epifluorescence. Recordings were made with Clampex 10.7 software running a Multiclamp 700B (Molecular Devices). Signals were digitized at 20 kHz and lowpass filtered at 4 kHz.

Data were collected at the resting membrane potential (RMP), then at −65 ± 3 mV. Unless stated otherwise, all data reported here were taken from recordings performed at −65 mV. Experiments were discontinued if series resistance rose to >30 MΩ or action potentials failed to overshoot 0 mV. The liquid junction potential was estimated to be 14.3 mV using Patchers Power Tools (IGORpro 7, Wavemetrics). The liquid junction potential was not corrected. The data sets were sampled from 40 WT and 42 *Fmr1* KO mice.

### 2.7 Electrophysiological properties

The analysis of electrophysiological data was performed as previously described (Lyuboslavsky et al., 2024). Briefly, RMP, membrane time constant (τ) and input resistance (*R*_N_) were measured from voltage responses to subthreshold current injections. Analyses were performed on a series of current injections ranging from −60 to +60 pA in 5 pA intervals for 1000 ms or from −250 to +350 in 25 pA intervals. Voltage sag is the difference between peak hyperpolarization and steady‐state voltage deflection in the trace with a peak hyperpolarization of −100 mV. After‐hyperpolarization is the difference between peak hyperpolarization and baseline membrane potential following the offset of the first current stimulus to have ≥12 action potentials. In Figure 3f,i and Figure 5c, neurons in the wild‐type ZD wash‐on experiments were analysed in a previous study (Lyuboslavsky et al., 2024).

### 2.8 Burst and tonic action potentials

We measured action potential threshold as the point at which the third derivative of the membrane potential exceeded 0.3 V/s^3^. Firing frequency measurements for tonic firing included sweeps in which no action potentials were fired; however, frequency measurements for burst action potentials excluded instances in which no action potentials were present. Accommodation was measured as the slope of the linear relationship between the interspike intervals (ISIs) for each successive action potential during the first current step to elicit ≥12 action potentials. Low‐threshold calcium spikes (LTS) were isolated with 0.5 µM TTX. Analysis of LTSs was performed on the first depolarizing step that elicited an LTS or on the hyperpolarizing step that reached −100 mV for rebound LTSs. α‐Wave EPSPs (αEPSPs) were generated using the equation $I_{t}=I_{\max}\;\times \frac{t}{\alpha }\times e^{-\alpha t}$, where $I_{t}$ is the current I at elapsed time t, $I_{\max}$ is the maximum current, and α is a constant representing the decay rate.

### 2.9 Statistics

We used the ‘sampsizepwr’ function in MATLAB to calculate sample sizes based on preliminary data. To detect a difference in membrane time constant of 25% with an SD of 20 ms, given α of 0.05 and power of 0.8, we estimated ≥16 cells per group. All quantifications are presented in Tables 1 and 2. The *n*‐values are presented as neurons/animals. Data compared with a two‐way ANOVA or fixed effects (type III) are reported as mean ± 95% confidence interval (CI). Data compared with Mann–Whitney or Wilcoxon tests are presented with all data points and the median with 95% CI. We defined α as *p* < 0.05. Effect size is expressed as η^2^ and calculated only when *p *< 0.05, and η^2^ effect sizes are defined as follows: small, 0.01; medium, 0.06; and large, 0.14 (Cohen, 1988). Quantifications were performed using custom‐written code in MATLAB 2022b (Mathworks). Statistical analyses were performed using Prism v.8.0.0 (GraphPad). Graphs were made using GraphPad Prism, and figures were made using Adobe Illustrator v.24.3.

**TABLE 1:** Descriptive statistics and test statistics of pairwise comparisons.

Comparison	Groups	Cells/animals (*n*/*n*)	Mean ± SD	Median [±95% CI]	Statistical test	Test statistic	*p*‐Value	η^2^	Median difference [±95% CI]
Resting *V*_m_ ^a^ (mV)	MD‐L^b^	39/29	−60.92 ± 6.85	−62 [−65/−58]	Mann–Whitney	*U* = 1129	0.875		−1 [−2/2]
	MD‐L *Fmr1* KO^c^	59/38	−60.94 ± 6.82	−63 [−65/−61]					
Resting *V*_m_ (mV)	MD‐M^d^	20/14	−62.2 ± 8.46	−63.5 [−69/−61]	Mann–Whitney	*U* = 237	0.088		3.5 [−1/8]
	MD‐M *Fmr1* KO	33/21	−58.79 ± 8.17	−60 [−63/059]					
τ^e^ (ms)	MD‐L	38/27	58.01 ± 23.17	52.81 [46.51/63.34]	Mann–Whitney	*U* = 1070	0.925		1.45 [−6.96/6.58]
	MD‐L *Fmr1* KO	57/36	55.69 ± 16.54	54.27 [50.31/57.68]					
τ (ms)	MD‐M	20/13	68.35 ± 19.00	66.85 [60.25/78.31]	Mann–Whitney	*U* = 310	0.859		0.47 [−10.59/12.32]
	MD‐M *Fmr1* KO	32/19	69.36 ± 21.86	67.32 [59.18/80.89]					
*R*_N_ ^f^ (MΩ)	MD‐L	36/28	250.8 ± 115.7	214.2 [180.2/287.1]	Mann–Whitney	*U* = 679.5	0.0348	0.033	76.30 [4.00/110.7]
	MD‐L *Fmr1* KO	48/33	309.6 ± 135.5	290.5 [243.7/332.5]					
*R*_N_ (MΩ)	MD‐M	20/13	418.8 ± 189.9	397.2 [311.7/487.3]	Mann–Whitney	*U* = 230	0.183		66.55 [−42.7/159.2]
	MD‐M *Fmr1* KO	27/17	462.8 ± 163.0	472.6 [331.8/610.4]					
Voltage sag at −100 mV (mV)	MD‐L	40/30	−10.27 ± 4.86	−10.07 [−12.89/−7.96]	Mann–Whitney	*U* = 791	0.0037	0.08	3.19 [1.03/4.95]
	MD‐L *Fmr1* KO	60/39	−7.48 ± 4.22	−6.88 [−8.66/−5.84]					
Voltage sag at −100 mV (mV)	MD‐M	18/12	−5.45 ± 3.98	−4.52 [−7.5/−2.94]	Mann–Whitney	*U* = 237	0.242		1.71 [−0.66/2.88]
	MD‐M *Fmr1* KO	33/21	−3.95 ± 2.61	−2.81 [−5.41/−2.14]					
*R*_N_ (MΩ), ZD^g^ wash‐on	MD‐L pre	10/7	333.0 ± 133.0	355 [190/480]	Wilcoxon	*W* = 52	0.0059	3.50	235 [70/410]
	MD‐L post	10/7	547.0 ± 191.8	555 [330/720]					
*R*_N_ (MΩ), ZD wash‐on	MD‐L *Fmr1* KO pre	9/5	451.1 ± 129.6	460 [380/550]	Wilcoxon	*W* = 45	0.0039	3.16	70 [30/270]
	MD‐L *Fmr1* KO post	9/5	571.1 ± 156.2	580 [500/670]					
Voltage sag at −100 mV (mV), ZD wash‐on	MD‐L pre	11/7	−10.43 ± 3.30	−9.09 [−14.96/−7.04]	Wilcoxon	*W* = 66	0.0010	3.13	8.95 [7.15/13.90]
	MD‐L post	11/7	−0.32 ± 0.495	−0.13 [−1.06/0.06]					
Voltage sag at −100 mV (mV), ZD wash‐on	MD‐L *Fmr1* KO pre	8/5	−4.41 ± 3.02	−4.48 [−8.31/−0.807]	Wilcoxon	*W* = 36	0.0078	4.55	3.96 [0.77/8.41]
	MD‐L *Fmr1* KO post	8/5	−0.283 ± 0.293	−0.242 [−0.66/0.096]					
After‐hyperpolarization (mV)	MD‐L	41/31	−11.96 ± 5.92	−11.7 [−15.43/−9.24]	Mann–Whitney	*U* = 828	0.0360	0.05	2.17 [0.2/5.54]
	MD‐L *Fmr1* KO	54/37	−9.14 ± 5.85	−9.52 [−11.03/−6.39]					
After‐hyperpolarization (mV), ZD wash‐on	MD‐L pre	11/8	−13.74 ± 4.07	−13.73 [−17.88/−8.75]	Wilcoxon	*W* = 66	0.0010	3.13	16.21 [11.94/22.10]
	MD‐L post	11/8	3.38 ± 3.64	4.35 [−0.82/7.32]					
After‐hyperpolarization (mV), ZD wash‐on	MD‐L *Fmr1* KO pre	9/5	−7.62 ± 6.66	−6.99 [−17.81/−1.14]	Wilcoxon	*W* = 45	0.0039	3.16	10.4 [0.52/17.78]
	MD‐L *Fmr1* KO post	9/5	0.42 ± 1.83	−0.029 [−1.21/1.86]					
Rebound spike timing	MD‐L	39/29	33.29 ± 18.21	29.2 [24.25/31.4]	Mann–Whitney	*U* = 619.5	0.0014	0.13	10.03 [3.75/16.54]
	MD‐L *Fmr1* KO	52/36	45.6 ± 23.47	39.23 [33.5/46.5]					
Rebound spike timing, ZD wash‐on	MD‐L pre	9/6	29.81 ± 5.53	29.95 [24.3/35.65]	Wilcoxon	*W* = 45	0.0039	3.16	102.5 [51.9/180.8]
	MD‐L post	9/6	153.3 ± 100.0	123.6 [83.45/211.1]					
Rebound spike timing, ZD wash‐on	MD‐L *Fmr1* KO pre	9/5	67.58 ± 28.58	63.2 [75.0/211.7]	Wilcoxon	*W* = 29	0.0977		29.0 [−15.15/117.7]
	MD‐L *Fmr1* KO post	9/5	122.7 ± 86.91	86.8 [34.15/282]					
Accommodation index, tonic firing: RMP	MD‐L	9/9	0.002 ± 0.0022	0.0016 [0.00035/0.0037]	Mann–Whitney	*U* = 14	0.298		0.0009 [−0.001/0.003]
	MD‐L *Fmr1* KO	5/5	0.003 ± 0.0014	0.0026 [0.0014/0.0051]					
Action potential threshold, tonic firing: RMP (mV)	MD‐L	9/9	−32.82 ± 2.97	−33.07 [−36.25/−30.52]	Mann–Whitney	*U* = 19	0.699		−2.8 [−7.24/6.09]
	MD‐L *Fmr1* KO	5/5	−33.63 ± 5.36	−35.87 [−38.28/−26.98]					
Time to first spike, tonic firing: RMP (ms)	MD‐L	9/9	216 ± 230.3	147 [11/578]	Mann–Whitney	*U* = 19	0.943		−24 [−414/153]
	MD‐L *Fmr1* KO	5/5	214.2 ± 231.6	123 [50/622]					
Accommodation Index, tonic firing: mibefradil	MD‐L	8/6	0.0045 ± 0.0041	0.004 [0.00047/0.014]	Mann–Whitney	*U* = 12	0.284		−0.0011 [−0.005/0.001]
	MD‐L *Fmr1* KO	5/4	0.0024 ± 0.0014	0.002 [0.0009/0.0044]					
Action potential threshold, tonic firing: mibefradil (mV)	MD‐L	8/6	−37.07 ± 4.56	−37.66 [−42.27/−27.59]	Mann–Whitney	*U* = 9.5	0.1368		4.46 [−1.56/8.03]
	MD‐L *Fmr1* KO	5/4	−33.98 ± 2.82	−33.2 [−37.57/−31.03]					
Time to first spike, tonic firing: mibefradil (ms)	MD‐L	8/6	114 ± 55.31	99 [47/190]	Mann–Whitney	*U* = 14	0.9307		−1 [−92/176]
	MD‐L *Fmr1* KO	5/4	136 ± 98.92	98 [36/292]					
Burst threshold (mV)	MD‐L	30/22	−38.99 ± 3.57	−39.69 [−40.77/−37.75]	Mann–Whitney	*U* = 550	0.8085		1.17 [−1.67/2.16]
	MD‐L *Fmr1* KO	38/28	−39.02 ± 4.62	−38.52 [−40.74/−37.66]					
Step burst LTS spike threshold (mV)	MD‐L	9/5	−48.14 ± 2.91	−48.43 [−50.84/−45.56]	Mann–Whitney	*U* = 24	0.4698		−1.36 [−4.34/1.39]
	MD‐L *Fmr1* KO	7/5	−49.59 ± 1.96	−49.9 [−52.05/−47.15]					
Step burst LTS peak time (ms)	MD‐L	9/5	87.67 ± 20.41	89 [68/110]	Mann–Whitney	*U* = 24	0.4698		13 [−25/44]
	MD‐L *Fmr1* KO	7/5	98.71 ± 31.11	102 [58/136]					
Step burst LTS threshold time (ms)	MD‐L	9/5	62.78 ± 22.79	64 [41/90]	Mann–Whitney	*U* = 21.5	0.312		5 [−21/54]
	MD‐L *Fmr1* KO	7/5	78.14 ± 30.45	69 [38/118]					
Rebound AP threshold (mV)	MD‐L	33/24	−41.07 ± 3.045	−41.75 [−42.88/−40.56]	Mann–Whitney	*U* = 607.5	0.365		1.8 [−0.92/2.63]
	MD‐L *Fmr1* KO	42/31	−40.16 ± 4.88	−39.95 [−41.9/−38.6]					
Rebound LTS spike threshold (mV)	MD‐L	12/7	−56.54 ± 2.62	−56.39 [−59.45/−54.66]	Mann–Whitney	*U* = 57	0.872		−0.71 [−2.4/2.45]
	MD‐L *Fmr1* KO	10/5	−56.54 ± 2.18	−57.09 [−58.57/−53.6]					
Rebound LTS peak time (ms)	MD‐L	12/7	50.58 ± 21.05	48.5 [30/69]	Mann–Whitney	*U* = 40	0.197		9.5 [−6/37]
	MD‐L *Fmr1* KO	10/5	64.8 ± 24.99	58 [41/90]					
Rebound LTS threshold time (ms)	MD‐L	12/7	45.58 ± 25.75	43.5 [24/59]	Mann–Whitney	*U* = 27	0.0284	0.22	15.5 [1/68]
	MD‐L *Fmr1* KO	10/5	77.4 ± 36.04	59 [47/127]					
Accommodation index, tonic firing: MD‐M	MD‐M	4/4	0.0018 ± 0.0014	0.002 [0.00004/0.0029]	Mann–Whitney	*U* = 8	0.730		−0.0008 [−0.003/0.004]
	MD‐M *Fmr1* KO	5/5	0.0016 ± 0.0021	0.0013 [−0.00022/0.0049]					
Action potential threshold, tonic firing: MD‐M (mV)	MD‐M	4/4	−32.58 ± 5.52	−33.1 [−37.41/−26.7]	Mann–Whitney	*U* = 7	0.556		2.19 [07.23/15.18]
	MD‐M *Fmr1* KO	5/5	−29.85 ± 5.50	−30.91 [−36.27/−21.98]					
Time to first spike, tonic firing: MD‐M (ms)	MD‐M	4/4	196.3 ± 124.7	187 [57/354]	Mann–Whitney	*U* = 9	0.9048		−68 [−248/468]
	MD‐M *Fmr1* KO	5/5	243.4 ± 238.9	119 [37/622]					
Burst action potential threshold (mV)	MD‐M	16/11	−36.09 ± 4.27	−36.88 [−38.27/−35.25]	Mann–Whitney	*U* = 140.5	0.159		−1.57 [−3.53/0.63]
	MD‐M *Fmr1* KO	24/14	−38.28 ± 4.11	−38.45 [−40.23/−35.06]					
Rebound action potential threshold (mV)	MD‐M	18/12	−37.51 ± 3.54	−38.5 [−39.42/−36.57]	Mann–Whitney	*U* = 185	0.185		−1.02 [−3.22/0.61]
	MD‐M *Fmr1* KO	27/17	−39.11 ± 3.16	−39.52 [−40.99/−37.67]					
First αEPSP amplitude	MD‐L	12/10	1.85 ± 0.769	1.9 [1.13/2.11]	Mann–Whitney	*U* = 52	0.616		−0.12 [−0.42/0.73]
	MD‐L *Fmr1* KO	10/10	1.94 ± 0.540	1.78 [1.46/2.7]					
*R*_N_ (MΩ), ZD wash‐on difference (post–pre)	MD‐L	10/7	214.0 ± 150.6	235 [70/410]	Mann–Whitney	*U* = 24.5	0.0989		−165 [−220/50]
	MD‐L *Fmr1* KO	9/5	120.0 ± 111.1	70 [30/270]					
Sag (mV), ZD wash‐on difference (post–pre)	MD‐L	11/7	10.11 ± 2.99	8.95 [7.15/13.9]	Mann–Whitney	*U* = 7	0.0012	0.49	−4.99 [−9.37/−2.30]
	MD‐L *Fmr1* KO	8/5	4.13 ± 3.09	3.96 [0.77/8.41]					
AHP (mV), ZD wash‐on difference (post–pre)	MD‐L	11/8	17.11 ± 4.00	16.21 [11.94/22.10]	Mann–Whitney	*U* = 18	0.0159	0.29	−10.42 [−15.69/−2.07]
	MD‐L *Fmr1* KO	9/5	8.04 ± 7.64	5.78 [0.52/17.78]					
RST (ms), ZD wash‐on difference (post–pre)	MD‐L	9/6	129.7 ± 96.7	104.3 [68.25/180.8]	Mann–Whitney	*U* = 17	0.0400	0.24	−75.30 [−130.0/−16.35]
	MD‐L *Fmr1* KO	9/5	55.19 ± 91.54	29 [−15.15/118.0]					

^a^ membrane voltage

^b^ lateral subnucleus of the mediodorsal thalamus

^c^ knockout

^d^ medial subnucleus of the mediodorsal thalamus

^e^ membrane time constant

^f^ input resistance

^g^ ZD7288, HCN channel blocker

**TABLE 2:** ANOVA and fixed effects statistics tables for experiments with multiple measurement points.

Voltage Sag (mV)						Rebound spike timing (ms)		
**ANOVA table**	**SS**	**DF**	**MS**	***F*(DFn, DFd)**	***p*‐Value**	**Fixed effects (type III)**	***F*(DFn, DFd)**	***p*‐Value**
Interaction	119.1	9	13.23	*F*(9, 747) = 4.862	*p *< 0.0001	Current (pA)	*F*(1.962, 189.9) = 120.2	<0.0001
Current (pA)	3796	9	421.8	*F*(1.122, 93.16) = 155.0	*p *< 0.0001	Genotype	*F*(1, 111) = 6.123	0.0149
Genotype	730.6	1	730.6	*F*(1, 83) = 7.133	*p* = 0.0091	Interaction	*F*(9, 871) = 1.462	0.1578
Neuron	8501	83	102.4	*F*(83, 747) = 37.64	*p *< 0.0001			
Residual	2033	747	2.721			**Rebound spike timing (ms) WT** b **Rebound spike timing (ms) WT** b **± ZD** ** ± ZD** ^c^		
						**Fixed effects (type III)**	***F*(DFn, DFd)**	***p*‐Value**
**50 Hz αEPSP** **a** **summation**						Current (pA)	*F*(9, 142) = 5.538	<0.0001
**ANOVA table**	**SS**	**DF**	**MS**	***F*(DFn, DFd)**	***p*‐Value**	Genotype	*F*(1, 16) = 12.40	0.0028
Interaction	2.366	9	0.2629	*F*(9, 180) = 1.604	*p* = 0.1168	Interaction	*F*(9, 142) = 1.101	0.366
αEPSP number	62.11	9	6.901	*F*(1.121, 22.42) = 42.12	*p *< 0.0001			
Genotype	6.054	1	6.054	*F*(1, 20) = 1.543	*p* = 0.2285	**Rebound Spike timing (ms) F*mr1* KO** **d** ** ± ZD**		
Neuron	78.47	20	3.923	*F*(20, 180) = 23.95	*p *< 0.0001	**Fixed effects (type III)**	***F*(DFn, DFd)**	***p*‐Value**
Residual	29.49	180	0.1638			Current (pA)	*F*(1.163, 16.15) = 3.816	0.0633
						Genotype	*F*(1, 15) = 3.478	0.0819
**100 Hz αEPSP summation**						Interaction	*F*(9, 125) = 3.230	0.0015
**ANOVA table**	**SS**	**DF**	**MS**	***F*(DFn, DFd)**	***p*‐Value**			
Interaction	2.107	9	0.2341	*F*(9, 180) = 0.7943	*p* = 0.6219	**ISI** **e** **tonic firing: RMP** ^f^		
αEPSP number	203	9	22.56	*F*(1.040, 20.80) = 76.54	*p *< 0.0001	**Fixed effects (type III)**	***F*(DFn, DFd)**	***p*‐Value**
Genotype	2.646	1	2.646	*F*(1, 20) = 0.3927	*p* = 0.5380	Spike number	*F*(3.817, 53.06) = 5.416	0.0012
Neuron	134.8	20	6.738	*F*(20, 180) = 22.86	*p *< 0.0001	Genotype	*F*(3, 19) = 0.1522	0.927
Residual	53.05	180	0.2947					
						**ISI tonic firing: Mibefradil**		
**200 Hz αEPSP summation**						**Fixed effects (type III)**	***F*(DFn, DFd)**	***p*‐Value**
**ANOVA table**	**SS**	**DF**	**MS**	***F*(DFn, DFd)**	***p*‐Value**	Spike number	*F*(3.259, 30.48) = 4.344	0.01
Interaction	2.135	9	0.2373	*F*(9, 180) = 0.5865	*p* = 0.8071	Genotype	*F*(1, 11) = 1.426	0.2576
αEPSP number	432.4	9	48.04	*F*(1.013, 20.26) = 118.8	*p *< 0.0001			
Genotype	3.878	1	3.878	*F*(1, 20) = 0.4967	*p* = 0.4891	**Action potentials per burst**		
Neuron	156.1	20	7.807	*F*(20, 180) = 19.30	*p *< 0.0001	**Fixed effects (type III)**	***F*(DFn, DFd)**	***p*‐Value**
Residual	72.82	180	0.4045			Currrent (pA)	*F*(12, 454) = 22.30	<0.0001
						Genotype	*F*(1, 67) = 0.4362	0.5112
**αEPSP summation**								
**ANOVA table**	**SS**	**DF**	**MS**	***F*(DFn, DFd)**	***p*‐value**	**Action potentials per rebound burst**		
Interaction	0.8962	2	0.4481	*F*(2, 40) = 0.3779	*p* = 0.6877	**Fixed effects (type III)**	***F*(DFn, DFd)**	***p*‐Value**
αEPSP frequency	90.52	2	45.26	*F*(1.992, 39.84) = 38.17	*p *< 0.0001	Current (pA)	*F*(12.00, 487.0) = 24.96	<0.0001
Genotype	3.141	1	3.141	*F*(1, 20) = 0.5610	*p* = 0.4626	Genotype	*F*(1, 69) = 2.270	0.1365
Neuron	112	20	5.599	*F*(20, 40) = 4.721	*p *< 0.0001	Interaction	*F*(12, 487) = 0.8202	0.6296
Residual	47.43	40	1.186					
						**ISI tonic firing: MD‐M** ^g^		
**Tonic firing: RMP**						**Fixed effects (type III)**	***F*(DFn, DFd)**	***p*‐Value**
**ANOVA table**	**SS**	**DF**	**MS**	***F*(DFn, DFd)**	***p*‐Value**	Spike number	*F*(2.093, 13.26) = 3.044	0.0799
Interaction	235	14	16.79	*F*(14, 168) = 0.1284	*p *> 0.9999	Genotype	*F*(1, 7) = 0.1590	0.702
Current (pA)	55210	14	3944	*F*(1.568, 18.81) = 30.17	*p *< 0.0001			
Genotype	3.315	1	3.315	*F*(1, 12) = 0.001586	*p* = 0.9689	**Action potentials per burst: MD‐M**		
Neuron	25084	12	2090	*F*(12, 168) = 15.99	*p *< 0.0001	**Fixed effects (type III)**	***F*(DFn, DFd)**	***p*‐Value**
Residual	21963	168	130.7			Currrent (pA)	*F*(12.00, 263.0) = 12.86	<0.0001
						Genotype	*F*(1, 46) = 1.021	0.3176
**Tonic firing: Mibefradil**						Interaction	*F*(12, 263) = 1.492	0.1267
**ANOVA table**	**SS**	**DF**	**MS**	***F*(DFn, DFd)**	***p*‐value**			
Interaction	919.1	14	65.65	*F*(14, 154) = 0.4164	*p* = 0.9679	**Action potentials per rebound burst: MD‐M**		
Current (pA)	13626	14	973.3	*F*(1.366, 15.03) = 6.174	*p* = 0.0181	**Fixed effects (type III)**	***F*(DFn, DFd)**	***P*‐value**
Genotype	283.1	1	283.1	*F*(1, 11) = 0.1956	*p* = 0.6668	Current (pA)	*F*(12, 368) = 14.26	<0.0001
Neuron	15918	11	1447	*F*(11, 154) = 9.179	*p *< 0.0001	Genotype	*F*(1, 49) = 0.01557	0.9012
Residual	24278	154	157.7					
								
**Voltage Sag**: **MD‐M (mV)**								
**ANOVA table**	**SS**	**DF**	**MS**	***F*(DFn, DFd)**	***p*‐Value**			
Interaction	40.13	9	4.458	*F*(9, 369) = 3.235	*p* = 0.0009			
Current (pA)	486	9	54	*F*(1.201, 49.24) = 39.18	*p *< 0.0001			
Genotype	191	1	191	*F*(1, 41) = 3.165	*p* = 0.0826			
Neuron	2474	41	60.34	*F*(41, 369) = 43.78	*p *< 0.0001			
Residual	508.6	369	1.378					
								
**Tonic firing: MD‐M**								
**ANOVA table**	**SS**	**DF**	**MS**	***F*(DFn, DFd)**	***p*‐Value**			
Interaction	1664	14	118.8	*F*(14, 84) = 3.218	*p* = 0.0004			
Current (pA)	19664	14	1405	*F*(1.402, 8.411) = 38.03	*p* = 0.0001			
Genotype	420.5	1	420.5	*F*(1, 6) = 1.005	*p* = 0.3549			
Neuron	2511	6	418.6	*F*(6, 84) = 11.33	*p *< 0.0001			
Residual	3103	84	36.94					

^a^ simultated excitatory post synaptic potential

^b^ wildtype

^c^ ZD7288, HCN channel blocker

^d^ knockout

^e^ interspike interval

^f^ resting membrane potential

^g^ medial subnucleus of the mediodorsal thalamus

## 3 RESULTS

**FIGURE 1:**
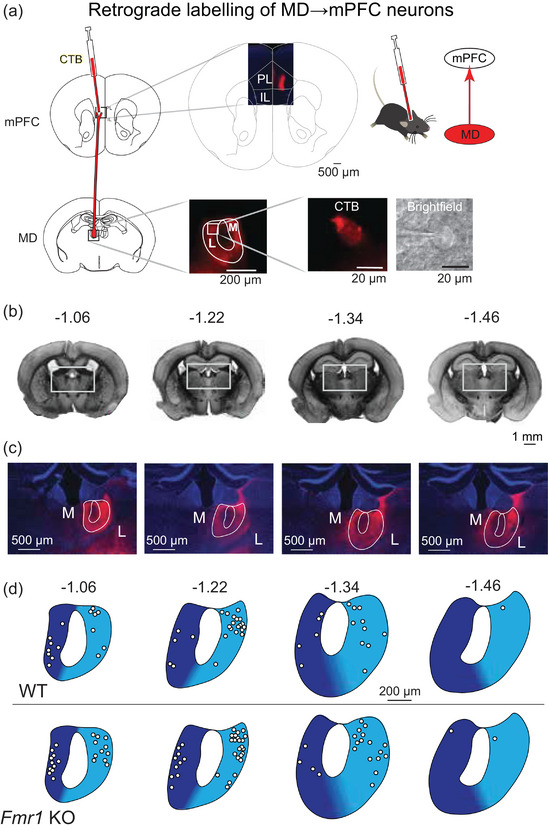
CTB injections in mPFC label MD‐L and MD‐M neurons that project to the mPFC. (a) Adult wild‐type (WT) and *Fmr1* KO mice were stereotaxically injected with the retrograde fluorescent tracer CTB into the mPFC to target prelimbic and infralimbic cortex. Fluorescently labelled MD→mPFC neurons were identified visually for patch‐clamp electrophysiology experiments. (b) Bright‐field photomicrographs of coronal mouse brain slices with the estimated distance from bregma (in millimetres) where MD→mPFC neurons were patched in this investigation. (c) Photomicrographs (×10) of the boxed areas from (b) demonstrating MD→mPFC labelled neurons (red) in each of the representative slices. (d) Map of the approximate locations of recorded neurons reported in this manuscript placed on a single representative atlas drawing for each coronal slice. MD‐M is dark blue. MD‐L is highlighted in cyan. CTB: cholera toxin, subunit B; mPFC: medial prefrontal cortex; MD‐L: mediodorsal thalamus, lateral subnucleus; MD‐M: mediodorsal thalamus, medial subnucleus.

**FIGURE 2:**
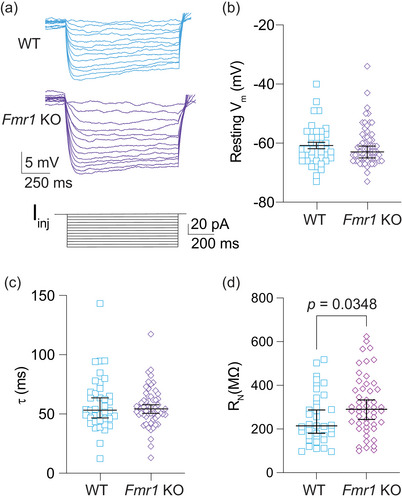
Greater *R*_N_ in *Fmr1* KO MD neurons compared with WT. (a) Representative traces showing voltage deflections in response to hyperpolarizing current steps ranging from 0 to −60 pA in 5 pA increments. (b) Resting *V*_m_ in WT and *Fmr1* KO neurons. *n*‐values: WT = 39/29, *Fmr1* KO = 59/38. Mann–Whitney *U*‐test: *p *= 0.875. (c) Membrane τ of WT and *Fmr1* KO neurons. *n*‐values: WT = 38/27, *Fmr1* KO = 57/36. Mann–Whitney *U*‐test: *p *= 0.925. (d) *R*_N_ in WT and *Fmr1* KO MD neurons measured from the linear portion of the *I–V* plot for each neuron. *n*‐values: WT = 36/28, *Fmr1* KO = 48/33. Mann–Whitney *U*‐test: *p *= 0.0348. RN: input resistance; KO: knockout; MD: mediodorsal thalamus; WT: wildtype; Vm: membrane potential; I‐V plot: voltage as a function of current plot.

**FIGURE 3:**
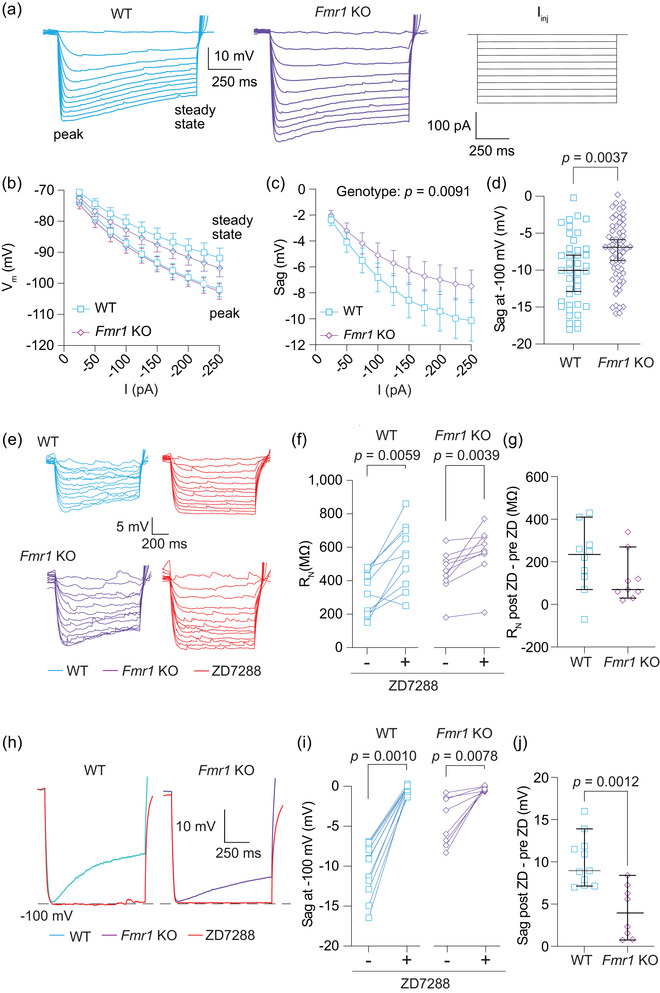
*Fmr1* KO MD neurons exhibit decreased HCN channel activity compared with WT neurons. (a) Representative traces of voltage responses from WT and *Fmr1* KO neurons to current stimuli ranging from 0 to −250 pA in 25 pA intervals. (b) *I–V* plot of WT and *Fmr1* KO neurons measured from both the peak and steady‐state voltage deflections. (c) Sag, measured as the difference between peak and steady‐state voltage deflections, in response to current stimuli from −25 to −250 pA in 25 pA intervals. *n*‐values: WT = 37/28, *Fmr1* KO = 48/34. Two‐way ANOVA, effect of genotype: *p *= 0.0091. (d) Sag in WT and *Fmr1* KO neurons when peak hyperpolarization reached −100 mV. *n*‐values: WT = 40/30, *Fmr1* KO = 60/39. Mann–Whitney *U*‐test: *p *= 0.0037. (e) Representative traces showing WT and *Fmr1* KO neuron voltage responses to current stimuli from 0 to −60 pA in 5 pA intervals before and after the application of 20 µM ZD7288. The linear portions of neuron *I–V* curves were used to calculate *R*_N_. (f) Before and after plots of the effect of ZD7288 on WT and *Fmr1* KO *R*_N_. *n*‐values: WT = 10/7, *Fmr1* KO = 9/5. WT: Wilcoxon test, *p *= 0.0059. *Fmr1* KO: Wilcoxon test, *p *= 0.0039. (g) *R*_N_ difference plots of ZD wash on experiment for WT and *Fmr1* KO neurons. *n*‐values: WT = 10/7, *Fmr1* KO = 9/5. Mann–Whitney *U*‐test: *p *= 0.0989. (h) Representative traces showing sag before and after ZD7288 application in WT and *Fmr1* KO neurons. (i) Before and after plots showing the effect of ZD7288 on sag measurements in WT and *Fmr1* KO neurons. *n*‐values: WT = 11/7, *Fmr1* KO = 8/5. WT: Wilcoxon test: *p* = 0.0010. *Fmr1* KO: Wilcoxon test, *p *= 0.0078. (j) R_N_ difference plots of ZD wash on experiment for WT and *Fmr1* KO neurons. *n*‐values: WT = 11/7, *Fmr1* KO = 8/5. Mann–Whitney *U*‐test: *p *= 0.0012. KO: knockout; MD: mediodorsal thalamus; HCN: hyperpolarization‐activated, cyclic nucleotide‐gated; WT: wildtype; I‐V plot: voltage as a function of current plot; RN: input resistance

**FIGURE 4:**
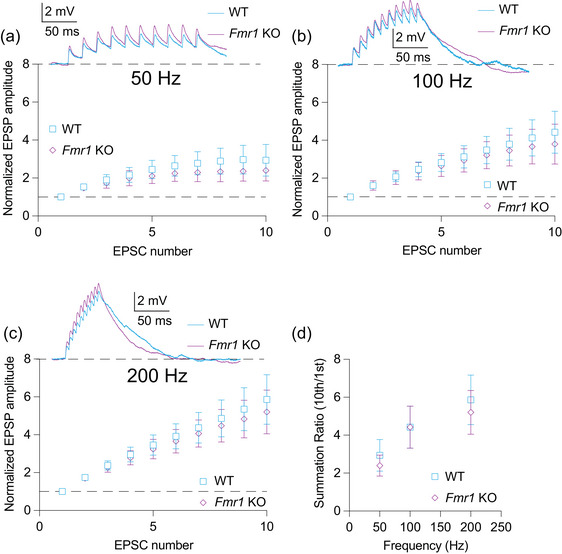
αEPSP summation is not different between WT and *Fmr1* KO MD neurons. (a) Normalized voltage response to αEPSPs delivered at 50 Hz in WT and *Fmr1* KO neurons. *n*‐values: WT = 12/10, *Fmr1* KO = 10/10. Two‐way ANOVA, effect of genotype: *p *= 0.229. (b) Normalized voltage response to αEPSPs delivered at 100 Hz in WT and *Fmr1* KO neurons. *n*‐values: WT = 12/10, *Fmr1* KO = 10/10. Two‐way ANOVA, effect of genotype: *p *= 0.538. (c) Normalized voltage response to αEPSPs delivered at 200 Hz in WT and *Fmr1* KO neurons. *n*‐values: WT = 12/10, *Fmr1* KO = 10/10. Two‐way ANOVA, effect of genotype: *p *= 0.489. (d) Summation ratio at 50, 100 and 200 Hz αEPSP rate in WT and *Fmr1* KO neurons. *n*‐values: WT = 12/10, *Fmr1* KO = 10/10. Two‐way ANOVA, effect of genotype: *p *= 0.463. αEPSP: modeled excitatory postsynaptic potential; WT: wildtype; KO: knockout; MD: mediodorsal thalamus

**FIGURE 5:**
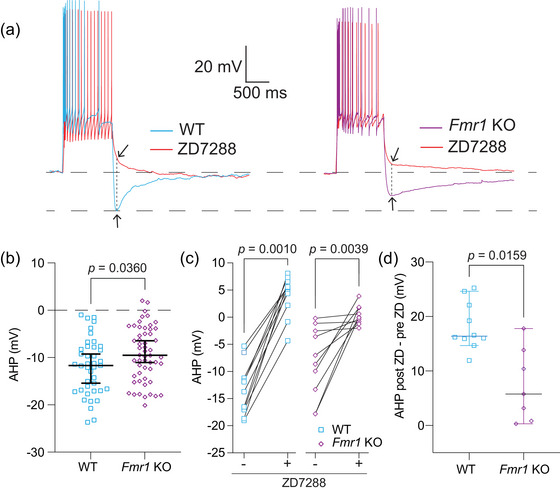
Decreased HCN activity results in decreased AHP amplitude in *Fmr1* KO neurons compared with WT control neurons. (a) Representative traces showing AHP amplitude in WT and *Fmr1* KO neurons before and after the application of 20 µM ZD7288. (b) AHP measured in WT and *Fmr1* KO neurons. *n*‐values: WT = 41/31, *Fmr1* KO = 54/37. Mann–Whitney *U*‐test: *p *= 0.0360. (c) Before and after plots of the effect of ZD7288 on AHP in WT and *Fmr1* KO neurons. *n*‐values: WT = 11/8, *Fmr1* KO = 9/5. WT, Wilcoxon test: *p *= 0.0010. *Fmr1* KO, Wilcoxon test: *p* = 0.0039. (d) AHP difference plots of ZD7288 wash on experiment for WT and *Fmr1* KO neurons. *n*‐values: WT = 11/8, *Fmr1* KO = 9/5. Mann–Whitney *U*‐test: *p *= 0.0159. HCN: hyperpolarization‐activated, cyclic‐nucleotide gated channels; AHP: afterhyperpolarization; KO: knockout; WT: wildtype

**FIGURE 6:**
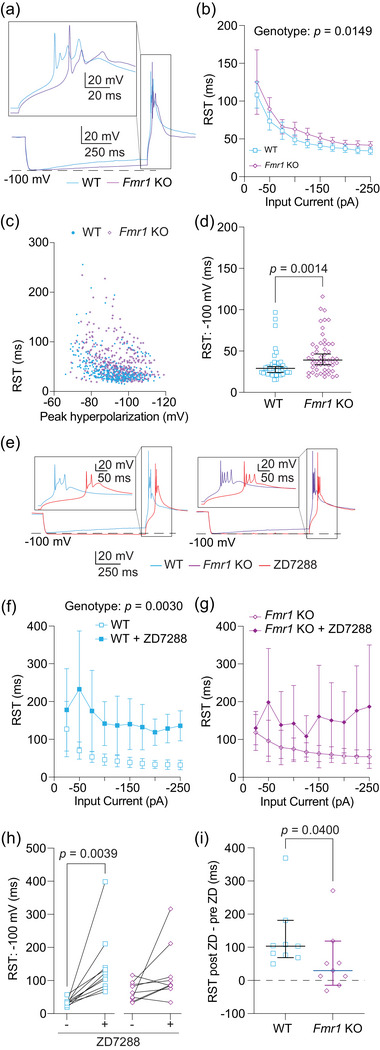
Rebound spike timing (RST) depends on HCN activity and is delayed in *Fmr1* KO mice.  (a) Representative traces of RST measured as the time from the offset of the hyperpolarizing current to the peak of the first action potential in the burst. Boxed region is expanded in the inset at the top of the panel. (b) RST measured at current stimuli ranging from 0 to −250 pA in 25 pA intervals. *n*‐values: WT = 39/29, *Fmr1* KO = 52/36. Mixed effects analysis, effect of genotype: *p *= 0.0149. (c) Plot of RST by the peak hyperpolarization reached in response to current stimuli. (d) RST measured when peak hyperpolarization reached −100 mV. *n*‐values: WT = 39/29, *Fmr1* KO = 52/36. Mann–Whitney *U*‐test: *p *= 0.0014. (e) Representative traces showing rebound bursts before and after the application of 20 µM ZD7288 in WT and *Fmr1* KO neurons. Insets represent expanded views of the boxed regions. (f) RST measurements in response to hyperpolarizing current stimuli from 0 to −250 pA in 25 pA intervals in WT MD neurons before and after wash‐on of 20 µM ZD7288. *n*‐values: WT = 9/6. WT, fixed effects (type III) analysis, effect of genotype: *p *= 0.0028. (g) RST measurements in response to hyperpolarizing current stimuli from 0 to −250 pA in 25 pA intervals in *Fmr1* KO MD neurons before and after wash‐on of 20 µM ZD7288. *n*‐values: *Fmr1* KO = 9/5. *Fmr1* KO, fixed effects (type III) analysis, effect of genotype: *p *= 0.0819. (h) Before and after plots of RST showing the effects of 20 µM ZD7288 wash‐on in WT and *Fmr1* KO neurons measured when peak hyperpolarization reached −100 mV. *n*‐values: WT = 9/6, *Fmr1* KO = 9/5. WT: Wilcoxon test, *p *= 0.0039. *Fmr1* KO: Wilcoxon test, *p* = 0.0977. (i) RST difference plots of ZD wash on experiment for WT and *Fmr1* KO neurons. *n*‐values: WT = 9/6, *Fmr1* KO = 9/5. Mann–Whitney *U*‐test: *p *= 0.0400. WT: wildtype; KO: knockout

**FIGURE 7:**
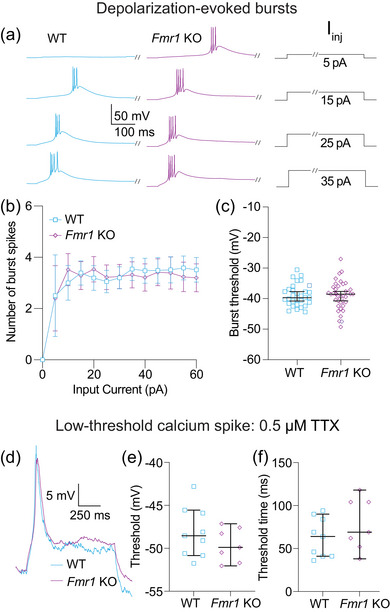
Bursting evoked by depolarizing current steps is not different between WT and *Fmr1* KO MD neurons. (a) Representative traces showing burst firing in response to 5, 15, 25 and 35 pA steps. Slashes on current steps show where voltage traces were truncated to show bursts better. (b) Number of action potentials per burst for current steps from 0 to 60 pA in 5 pA intervals. *n*‐values: WT = 30/22, *Fmr1* KO = 38/28. Fixed effects (type III) analysis, effect of genotype: *p *= 0.511. (c) Burst threshold measured from the first step to elicit a burst. *n*‐values: WT = 30/22, *Fmr1* KO = 38/28. Mann–Whitney *U*‐test: *p* = 0.8085. (d) Representative traces of LTS isolated by the application of 0.5 µM TTX. (e) LTS threshold measured from the first current step to evoke a spike. *n*‐values: WT = 9/5, *Fmr1* KO = 7/5. Mann–Whitney *U*‐test: *p *= 0.4698. (f) LTS time to threshold measured from the first current step to evoke a spike. *n*‐values: WT = 9/5, *Fmr1* KO = 7/5. Mann–Whitney *U*‐test: *p *= 0.312. WT: wildtype; KO: knockout; MD: mediodorsal thalamus; LTS: low‐threshold calcium spike; TTX: tetrodotoxin

**FIGURE 8:**
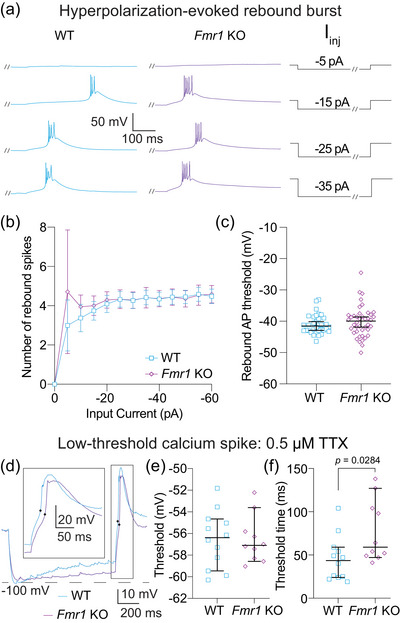
Bursting evoked by hyperpolarizing current steps is not different between WT and *Fmr1* KO MD neurons. (a) Representative traces showing burst firing in response to −5, −15, −25 and −35 pA steps. Slashes on current steps show where voltage traces were truncated to show bursts better. (b) Number of action potentials per burst for current steps from 0 to −60 pA in 5 pA intervals. *n*‐values: WT = 33/24, *Fmr1* KO = 42/31. Fixed effects (type III) analysis, effect of genotype: *p *= 0.137. (c) Burst threshold measured from the first hyperpolarizing step to elicit a burst. *n*‐values: WT = 33/24, *Fmr1* KO = 42/31. Mann–Whitney *U*‐test: *p* = 0.365. (d) Representative traces of LTSs isolated by the application of 0.5 µM TTX. (e) LTS threshold measured from the first hyperpolarizing current step to evoke a spike. *n*‐values: WT = 12/7, *Fmr1* KO = 10/5. Mann–Whitney *U*‐test: *p *= 0.872. Black dots represent threshold measurements for LTS. (f) LTS time to threshold measured from the first hyperpolarizing current step to evoke a spike. *n*‐values: WT = 12/7, *Fmr1* KO = 10/5. Mann–Whitney *U*‐test: *p *= 0.0284. WT: wildtype, KO: knockout, I_inj_: current injection, TTX: tetrodotoxin, LTS: low‐threshold calcium spike.

**FIGURE 9:**
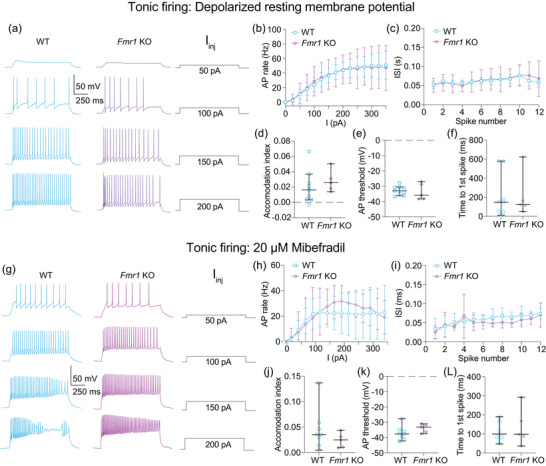
Tonic action potential firing is not different between WT and *Fmr1* KO MD neurons. (a–f) Tonic firing data collected from cells that did not fire bursts at resting membrane potential. (a) Representative traces showing tonic action potential firing in WT and *Fmr1* KO neurons in response to 50, 100, 150 and 200 pA current steps. (b) Action potential firing rate measured at current steps from 0 to 350 in 25 pA intervals. *n*‐values: WT = 9/9, *Fmr1* KO = 5/5. Two‐way ANOVA, effect of genotype: *p *= 0.969. (c) ISI of action potentials in the first step with ≥12 action potentials. *n*‐values: WT = 9/9, *Fmr1* KO =  = 5/5. Fixed effects (type III), effect of genotype: *p *= 0.927. (d) Accommodation index measurements taken from the first current step to elicit ≥12 action potentials. *n*‐values: WT = 9/9, *Fmr1* KO = 5/5. Mann–Whitney *U*‐test: *p *= 0.298. (e) Action potential threshold measured from the first step with ≥12 action potentials. *n*‐values: WT = 9/9, *Fmr1* KO = 5/5. Mann–Whitney *U*‐test: *p *= 0.699. (f) Time to the peak of the first spike in the first current step to elicit one or more action potentials. *n*‐values: WT = 9/9, *Fmr1* KO = 5/5. Mann–Whitney *U*‐test: *p *= 0.943. (g–l) Tonic firing data collected from cells held at −65 mV with the Ca_V_3 blocker mibefradil added to prevent bursting. (g) Representative traces showing tonic action potential firing in WT and *Fmr1* KO neurons in response to 50, 100, 150 and 200 pA current steps. (h) Action potential firing rate measured at current steps from 0 to 350 in 25 pA intervals. *n*‐values: WT = 8/6, *Fmr1* KO = 5/4. Two‐way ANOVA, effect of genotype: *p *= 0.667. (i) ISI of action potentials in the first step with ≥12 action potentials. *n*‐values: WT = 8/6, *Fmr1* KO = 5/4. Fixed effects (type III) analysis, effect of genotype: *P *= 0.258. (j) Accommodation index measurements taken from the first current step to elicit ≥12 action potentials. *n*‐values: WT = 8/6, *Fmr1* KO = 5/4. Mann–Whitney *U*‐test: *p *= 0.284. (k) Action potential threshold measured from the first step with ≥12 action potentials. *n*‐values: WT = 8/6, *Fmr1* KO = 5/4. Mann–Whitney *U*‐test: *p *= 0.137. (l) Time to the peak of the first spike in the first current step to elicit one or more action potentials. *n*‐values: WT = 8/6, *Fmr1* KO = 5/4. Mann–Whitney *U*‐test: *p *= 0.931. WT: wildtype, KO: knockout, AP: action potential, ISI: interspike interval, I_inj_: current injection.

**FIGURE 10:**
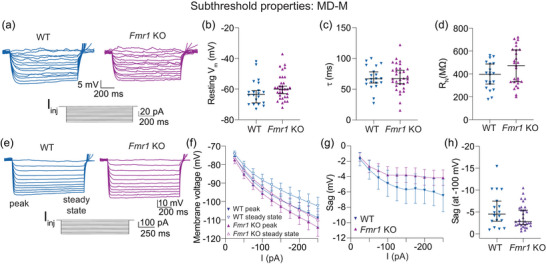
WT and *Fmr1* KO MD‐M neurons show no difference in subthreshold properties. (a) Representative traces showing voltage responses to hyperpolarizing current steps from 0 to −60 pA in 5 pA intervals in WT and *Fmr1* KO neurons. (b) Resting membrane potential in WT and *Fmr1* KO MD‐M neurons. *n*‐values: WT = 20/14, *Fmr1* KO = 33/21. Mann–Whitney *U*‐test: *p *= 0.088. (c) Membrane τ measured from a step to −10 pA in WT and *Fmr1* KO neurons. *n*‐values: WT = 20/13, *Fmr1* KO = 32/19. Mann–Whitney *U*‐test: *p *= 0.859. (d) Input resistance measurements from the linear portion of the *I–V* plot for WT and *Fmr1* KO MD‐M neurons. *n*‐values: WT = 20/13, *Fmr1* KO = 27/17. Mann–Whitney *U*‐test: *p *= 0.183. (e) Representative traces of voltage responses from WT and *Fmr1* KO MD‐M neurons to current stimuli ranging from 0 to −250 pA in 25 pA intervals. (f) *I–V* plot of WT and *Fmr1* KO neurons measured from both the peak and steady‐state voltage deflections. (g) Sag, measured as the difference between peak and steady‐state voltage deflections, in response to current stimuli from −25 to −250 pA in 25 pA intervals. *n*‐values: WT = 16/11, *Fmr1* KO = 27/17. Two‐way ANOVA, effect of genotype: *p *= 0.0826. (h) Sag in WT and *Fmr1* KO neurons when peak hyperpolarization reached −100 mV. *n*‐values: WT = 18/12, *Fmr1* KO = 33/21. Mann–Whitney *U*‐test: *p *= 0.242. WT: wildtype, KO: knockout, MD‐M: medial mediodorsal thalamus, I‐V: current ‐ voltage.

**FIGURE 11:**
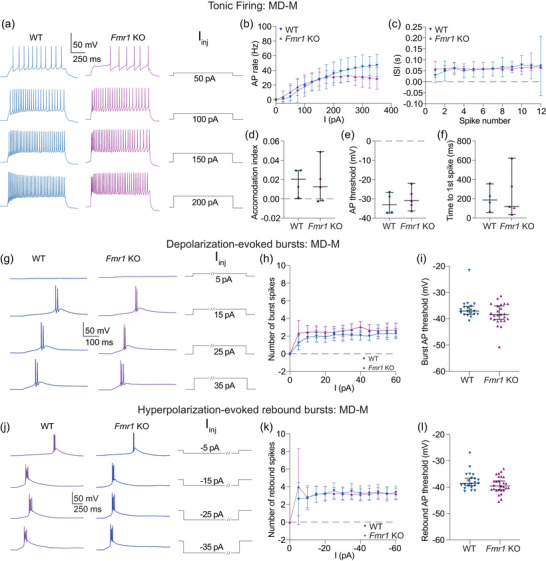
WT and *Fmr1* KO MD‐M neurons show no difference in suprathreshold properties. (a–f) Tonic firing data collected from cells that did not fire bursts at resting membrane potential. (a) Representative traces showing tonic action potential firing in WT and *Fmr1* KO MD‐M neurons in response to 50, 100, 150 and 200 pA current steps. (b) Action potential firing rate measured at current steps from 0 to 350 in 25 pA intervals. *n*‐values: WT = 4/4, *Fmr1* KO = 5/5. Two‐way ANOVA, effect of genotype: *p *= 0.355. (c) ISI of action potentials in the first step with ≥12 action potentials. *n*‐values: WT = 4/4, *Fmr1* KO = 5/5. Fixed effects (type III) analysis, effect of genotype: *p *= 0.702. (d) Accommodation index measurements taken from the first current step to elicit ≥12 action potentials. *n*‐values: WT = 4/4, *Fmr1* KO = 5/5. Mann–Whitney *U*‐test: *p *= 0.730. (e) Action potential threshold measured from the first step with ≥12 action potentials. *n*‐values: WT = 4/4, *Fmr1* KO = 5/5. Mann–Whitney *U*‐test: *p *= 0.556. (f) Time to the peak of the first spike in the first current step to elicit any action potentials. *n*‐values: WT = 4/4, *Fmr1* KO = 5/5. Mann–Whitney *U*‐test: *p *= 0.9048. (g) Representative traces showing burst firing in response to 5, 15, 25 and 35 pA steps. Slashes on current steps show where voltage traces were truncated to better visualize bursts. (h) Number of action potentials per burst for current steps from 0 to 60 pA in 5 pA intervals. *n*‐values: WT = 16/11, *Fmr1* KO = 24/14. Fixed effects (type III) analysis, effect of genotype: *p *= 0.318. (i) Burst threshold measured from the first step to elicit a burst. *n*‐values: WT = 16/11, *Fmr1* KO = 24/14. Mann–Whitney *U*‐test: *p *= 0.159. (j) Representative traces showing burst firing in response to −5, −15, −25 and −35 pA steps. Slashes on current steps show where voltage traces were truncated to better show bursts. (k) Number of action potentials per burst for current steps from 0 to −60 pA in 5 pA intervals. *n*‐values: WT = 18/12, *Fmr1* KO = 27/17. Fixed effects (type III) analysis, effect of genotype: *p *= 0.9012. (l) Burst threshold measured from the first hyperpolarizing step to elicit a burst. *n*‐values: WT = 18/12, *Fmr1* KO = 27/17. Mann–Whitney *U*‐test: *p *= 0.185. WT: wildtype, KO: knockout, ISI: interspike interval, MD‐M: medial mediodorsal thalamus, AP: action potential.

To investigate the intrinsic effects of *Fmr1* KO on MD neurons projecting to mPFC (‘MD→mPFC neurons’, but for simplicity referred to as ‘MD neurons’ in this manuscript), we made whole‐cell current‐clamp recordings of fluorescently labelled thalamocortical neurons in the MD region of the thalamus in acute *ex vivo* mouse brain slices (Figure 1a). MD neurons were identified visually and distinguished as medial (MD‐M) or lateral (MD‐L) based on the distance from the midline and anatomical landmarks (Figure 1b–d). Both MD‐M and MD‐L neurons in WT and *Fmr1* KO mice were investigated during this study; however, intrinsic properties differed only between WT and *Fmr1* KO MD‐L neurons. As a result, MD‐L data are presented in Figures 1, 2, 3, 4, 5, 6, 7, 8, 9, and all MD‐M data are presented in Figures 10 and 11.

### 3.1 *Fmr1* KO MD‐L neurons display greater input resistance compared with WT MD neurons

We measured subthreshold, intrinsic neuronal properties of WT and *Fmr1* KO fluorescently labelled neurons (Figure 2a). We found no difference in either RMP (Figure 2b; Mann–Whitney *U*‐test: *p* = 0.875) or membrane τ (Figure 2c; Mann–Whitney *U*‐ *U*‐test: *p *= 0.925) of *Fmr1* KO neurons compared with WT. We observed greater input resistance (*R*_N_) in *Fmr1* KO MD neurons compared with WT control neurons (Figure 2d; Mann–Whitney *U*‐test: *p *= 0.0348, η^2^ = 0.033). Based on previous reports of HCN expression in MD neurons (Lyuboslavsky et al., 2024; Notomi & Shigemoto, 2004) and extensive literature identifying modifications in HCN expression in *Fmr1* KO mouse models (Brager et al., 2012; Brandalise et al., 2020; Deng & Klyachko, 2016a, 2016b; Deng et al., 2021; Kalmbach et al., 2015), we next investigated intrinsic measures of HCN activity to assess potential differences in HCN function between WT and *Fmr1* KO MD neurons.

### 3.2 *Fmr1* KO MD‐L neurons have reduced sag when compared with WT MD neurons

We used voltage sag, a well‐established measure of HCN activity, to investigate differences in HCN function (Figure 3a). Figure 3b shows *V–I* plots of peak voltage deflection and steady‐state voltage deflection for both WT and *Fmr1* KO MD‐L neurons. Although both WT and *Fmr1* KO neurons exhibited clear sag in response to a range of current injections, voltage sag was reduced in *Fmr1* KO neurons compared with WT control neurons (Figure 3c; two‐way ANOVA, effect of genotype: *p *= 0.0091; η^2^ = 0.08). The difference reported in *R*_N_ in Figure 2d can affect comparisons of sag measured only by current injection. To account for differences in *R*_N_, we compared sag in WT and *Fmr1* KO neurons from a peak hyperpolarization of −100 mV. After normalizing for membrane hyperpolarization, the difference in sag persisted (Figure 3d; Mann–Whitney *U*‐test: *p *= 0.0037, η^2^ = 0.08). We used the HCN channel blocker ZD7288 (20 µM) to verify that the differences observed in intrinsic neuronal properties stem from HCN channel activity. The *R*_N_ increased significantly in both WT and *Fmr1* KO neurons with the application of ZD7288 (Figure 3e,f; Wilcoxon test, WT: *p *= 0.0059, η^2^ = 3.50; *Fmr1* KO: *p *= 0.0039, η^2^ = 3.16). We found no difference in the change in *R*_N_ attributable to the application of ZD7288 between WT and *Fmr1* KO MD neurons (Figure 3g; Mann–Whitney *U*‐test: *p *= 0.0989). We also measured the effect of ZD7288 on sag. As expected, sag was completely abolished by the wash‐on of ZD7288 in both WT and *Fmr1* KO MD neurons (; Wilcoxon test, WT: *p *= 0.0010, η^2^ = 3.13; *Fmr1* KO: *p* = 0.0078, η^2^ = 4.55). WT neurons showed a greater change in sag attributable to the application of ZD7288 compared with *Fmr1* KO neurons. This result provides further evidence that there is greater HCN activity in WT compared with *Fmr1* KO MD neurons (Figure 3j; Mann–Whitney *U*‐test: *p *= 0.0012, η^2^ = 0.49).

### 3.3 αEPSP summation is not different between WT and *Fmr1* KO MD‐L neurons

To test whether differences in HCN function between WT and *Fmr1* KO neurons affect signal summation, we injected trains of αEPSPs at frequencies of 50, 100 and 200 Hz. Stimulus amplitudes were adjusted to ∼1–2 mV. We found no difference in the amplitude of the first αEPSP injected between WT and *Fmr1* KO neurons (Mann–Whitney *U*‐test: *p *= 0.616). There was no difference in summation between WT and *Fmr1* KO neurons at 50 Hz (Figure 4a; two‐way ANOVA, effect of genotype: *p *= 0.229), 100 Hz (Figure 4b; two‐way ANOVA, effect of genotype: *p *= 0.538) or 200 Hz (Figure 4c; two‐way ANOVA, effect of genotype: *p *= 0.497). The summation ratio (10th αEPSP/first αEPSP) revealed no difference between WT and *Fmr1* KO neurons (Figure 4d; two‐way ANOVA, effect of genotype: *p *= 0.463). These results suggest that the differences in HCN channel function between WT and *Fmr1* KO neurons do not affect subthreshold summation of αEPSP inputs.

### 3.4 *Fmr1* KO MD neurons have greater AHP and delayed rebound spike timing compared with WT MD neurons

To investigate differences in AHP after a train of action potentials, we measured the minimum voltage amplitude reached within 500 ms of the offset of current injection. These measurements were performed both before and after the application of 20 µM ZD7288 (Figure 5a). Measurements of AHP showed a consistent difference in amplitude between WT and *Fmr1* KO neurons (Figure 5b; Mann–Whitney *U*‐test: *p *= 0.036, η^2^ = 0.05). The AHP was significantly reduced by the application of ZD7288 in both WT and *Fmr1* KO neurons (Figure 5c; Wilcoxon test, WT: *p* = 0.0010, η^2^ = 3.13; *Fmr1* KO: *p *= 0.0039, η^2^ = 3.16). We compared the effect of ZD7288 wash‐on (post‐ZD–pre‐ZD) and found that the magnitude of the change in AHP amplitude was greater in WT compared with *Fmr1* KO MD neurons (Figure 5d; Mann–Whitney *U*‐test: *p *= 0.0159, η^2^ = 0.29). These results suggest that the AHP is strongly influenced by HCN activity and has a reduced amplitude in *Fmr1* KO compared with WT neurons.

We next evaluated rebound spike timing (RST), defined as the time between the offset of the hyperpolarizing current step and the peak of the first action potential in a rebound burst (Figure 6a). RST measured against hyperpolarizing current step amplitude revealed a delay in RST in *Fmr1* KO neurons compared with WT control neurons [Figure 6b; fixed effects (type III) analysis, effect of genotype: *p *= 0.0149, η^2^ = 0.07]. To account for the peak level of depolarization, we plotted RST against peak hyperpolarization for WT and *Fmr1* KO MD neurons (Figure 6c). To account for the level of peak hyperpolarization attributable to differences in *R*_N_, we compared RST when peak hyperpolarization reached −100 mV in WT and *Fmr1* KO neurons and found that *Fmr1* KO neurons showed a greater delay in RST compared with WT control neurons (Figure 6d; WT: 33.29 ± 18.21 ms, *Fmr1* KO: 45.6 ± 23.47 ms; Mann–Whitney *U*‐test: *p *= 0.0014, η^2^ = 0.13).

In the absence of rebound burst firing, the release of hyperpolarization causes an HCN channel‐mediated depolarization before returning to rest (Mishra & Narayanan, 2025). We hypothesized that this effect contributes to the difference in RST observed here. To test HCN involvement, we measured RST before and after wash‐on of 20 µM ZD7288 (Figure 6e). When compared against current amplitude, WT [Figure 6f; WT fixed effects (type III) analysis, effect of genotype: *p *= 0.0028, η^2^ = 0.42] and not *Fmr1* KO [Figure 6g; *Fmr1* KO fixed effects (type III) analysis, effect of genotype: *p *= 0.0819] neurons showed a significant delay in RST with blockade of HCN channels. When normalized to steps in which peak hyperpolarization reached −100 mV, only WT neurons showed a significant difference in RST after ZD7288 wash‐on (Figure 6h; Wilcoxon test, WT: *p *= 0.0039, η^2^ = 3.16; *Fmr1* KO: *p *= 0.0977). We then compared the magnitude of the difference in RST attributable to ZD7288 wash‐on (post‐ZD–pre‐ZD) and found a significantly greater increase in RST in WT compared with *Fmr1* KO MD neurons (Figure 6i; Mann–Whitney *U*‐test: *p *= 0.0400, η^2^ = 0.24), suggesting that decreased HCN channel activity results in delayed RST in *Fmr1* KO neurons.

### 3.5 Burst firing and calcium spike properties are not different between WT and *Fmr1* KO MD neurons

Thalamic neurons display state‐dependent firing properties based on the availability of Ca_V_3 channels, whereby neurons fire tonic spikes when resting at depolarized potentials (when Ca_V_3 channels are inactivated) and fire bursts when hyperpolarized (when Ca_V_3 channels are available) (Llinás & Steriade, 2006). Figures 7, 8, 9 investigate differences in action potential firing based on neuron state and stimulus type.

We recorded bursts of action potentials in response to depolarizing current steps ranging from 0 to 60 pA in 5 pA intervals (Figure 7a). There were no differences in either the number of action potentials fired in each burst [Figure 7b; fixed effects (type III) analysis, effect of genotype: *p *= 0.511] or the threshold for the first action potential in the first step to elicit a burst (Figure 7c; Mann–Whitney *U*‐test: *p* = 0.8085). To compare calcium spikes evoked by depolarization, we applied the sodium channel blocker TTX (0.5 µM) (Figure 7d). We found no difference in LTS threshold (Figure 7e; Mann–Whitney *U*‐test: *p *= 0.4698) or in the time from the offset of the current injection to the peak of the LTS (Mann–Whitney *U*‐test: *p *= 0.4698) between WT and *Fmr1* KO neurons. Added variability from the slower activation time of Ca_V_3 channels compared with Na_V_ channels might obscure differences in LTS onset. To address this confound, we measured the latency from the onset of current to LTS threshold. We found no difference in spike timing using this measure (Figure 7f; Mann–Whitney *U*‐test: *p *= 0.312).

We then recorded the properties of rebound bursts evoked in response to hyperpolarizing current steps from 0 to −60 pA in 5 pA intervals (Figure 8a). There was no difference between WT and *Fmr1* KO neurons in the number of action potentials fired in each rebound burst [Figure 8b; fixed effects (type III) analysis, effect of genotype: *p *= 0.137] or the rebound burst threshold (Figure 8c; Mann–Whitney *U*‐test: *p* = 0.365). With the addition of TTX to isolate calcium spikes (Figure 8d) we found no difference in spike threshold (Figure 8e; Mann–Whitney *U*‐test: *p *= 0.872) or the timing between current offset and spike peak (Mann–Whitney *U*‐test: *p *= 0.197) between WT and *Fmr1* KO neurons. As in Figure 7f, we measured the time to LTS threshold to assess LTS latency using a more concise measure. We found that the latency to LTS threshold was greater in *Fmr1* KO compared with WT MD neurons after the offset of hyperpolarizing current (Figure 8f; WT: 45.58 ± 25.75, *Fmr1* KO: 77.4 ± 36.04; Mann–Whitney *U*‐test: *p *= 0.0284, η^2^ = 0.22). Our results show that waveform properties of rebound bursts and LTS associated with Ca_V_3 channels are not different between *Fmr1* KO and WT MD neurons. Consistent with measurements of RST in Figure 6, we identified greater latency to hyperpolarization‐evoked rebound LTS in *Fmr1* KO compared with WT MD neurons.

### 3.6 Tonic action potential firing is not different between WT and *Fmr1* KO MD neurons

To isolate tonic action potential firing, we took two approaches. First, we analysed cells that did not fire bursts at RMP (Figure 9a–f). Second, we held cells at −65 mV while blocking Ca_V_3 channels with 20 µM mibefradil, which prevents burst firing (Figure 9g–l). We used measures of firing frequency, ISI, spike threshold and accommodation to assess differences in tonic firing between WT and *Fmr1* KO MD neurons

No comparison of tonic firing properties at RMP was different between WT and *Fmr1* KO neurons (Figure 9a–f). We measured action potential firing rate (Figure 9b; two‐way ANOVA, effect of genotype: *p *= 0.969), ISI [Figure 9c; fixed effects (type III), effect of genotype: *p *= 0.927], accommodation (Figure 9d; Mann–Whitney *U*‐test: *p *= 0.298), action potential threshold (Figure 9e; Mann–Whitney *U*‐test: *p *= 0.699) and time to first action potential in the first depolarizing step to elicit an action potential (Figure 9f; Mann–Whitney *U*‐test: *p *= 0.943) and found no differences.

Likewise, no comparison of tonic firing properties during mibefradil wash‐on was different between WT and *Fmr1* KO neurons (Figure 9g–l). No difference was identified in measurements of action potential frequency (Figure 9h; two‐way ANOVA, effect of genotype: *p *= 0.667), ISI [Figure 9i; fixed effects (type III) analysis, effect of genotype: *p *= 0.258], accommodation (Figure 9j; Mann–Whitney *U*‐test: *p *= 0.284), action potential threshold (Figure 9k; Mann–Whitney *U*‐test: *p *= 0.1368) or the time to first action potential in the first current step to elicit an action potential (Figure 9l; Mann–Whitney *U*‐test: *p *= 0.9307). These results show that suprathreshold tonic firing is not affected in MD neurons in *Fmr1* KO animals.

### 3.7 Subthreshold properties are not different between WT and *Fmr1* KO MD‐M neurons

All data discussed previously came from the lateral subnucleus of the MD projecting to the mPFC (MD‐L→mPFC neurons). We tested whether these same findings were present in neurons in the medial subnucleus of the MD (MD‐M→mPFC neurons). We measured voltage responses to current stimuli from −60 to 60 pA in 5 pA intervals (Figure 10a) in WT and *Fmr1* KO MD‐M neurons. We found no differences in measurements of resting membrane potential (Figure 10b; Mann–Whitney *U*‐test: *p *= 0.088), membrane τ (Figure 10c; Mann–Whitney *U*‐test: *p *= 0.859) or *R*_N_ (Figure 10d; Mann–Whitney *U*‐test: *p *= 0.183). We also investigated the subthreshold property of sag to assess whether changes in HCN activity observed in MD‐L were also present in MD‐M. We recorded voltage responses ranging from −250 to 0 pA in 25 pA intervals in WT and *Fmr1* KO MD‐M neurons (Figure 10e). We created an *I–V* plot from the peak and steady‐state portions of voltage traces in response to each current step for both WT and *Fmr1* KO MD‐M neurons (Figure 10f) to calculate voltage sag at each current step. There was no difference in sag between WT and *Fmr1* KO MD‐M neurons either when measured at each current injection amplitude (Figure 10g; two‐way ANOVA, effect of genotype: *p *= 0.0826) or when traces with a peak hyperpolarization of −100 mV were analysed (Figure 10h; Mann–Whitney *U*‐test: *p *= 0.242).

### 3.8 Suprathreshold properties are not different between WT and *Fmr1* KO MD‐M neurons

We investigated tonic firing from WT and *Fmr1* KO MD‐M neurons that did not fire bursts at resting membrane potential (Figure 11a). Our experiments revealed no differences in action potential firing (Figure 11b; two‐way ANOVA, effect of genotype: *p *= 0.355) or ISI [Figure 11c; fixed effects (type III) analysis, effect of genotype: *p *= 0.702]. We found no differences in properties associated with changes in voltage‐gated channels associated with suprathreshold activity in WT and *Fmr1* KO MD‐M neurons, including accommodation (Figure 11d; Mann–Whitney *U*‐test: *p *= 0.730), action potential threshold (Figure 11e; Mann–Whitney *U*‐test: *p *= 0.556) and time to first action potential in the first step to elicit action potentials (Figure 11f; Mann–Whitney *U*‐test: *p *= 0.9048).

Burst firing was analysed in depolarizing current steps from 0 to 60 pA in 5 pA intervals (Figure 11g). No difference in either the number of action potentials per burst [Figure 11h; fixed effects (type III) analysis, effect of genotype: *p *= 0.3176] or in the burst action potential threshold (Figure 11i; Mann–Whitney *U*‐test: *p *= 0.159) was identified between WT and *Fmr1* KO MD‐M neurons.

Rebound burst firing was analysed after offset of hyperpolarizing current steps from −60 to 0 pA in 5 pA intervals (Figure 11j). No difference in either the number of action potentials per burst [Figure 11k; fixed effects (type III) analysis, effect of genotype: *p *= 0.9012] or in the rebound burst action potential threshold (Figure 11l; Mann–Whitney *U*‐test: *p *= 0.185) was identified between WT and *Fmr1* KO MD‐M neurons.

## 4 DISCUSSION

Reciprocal connectivity between MD and mPFC is required for higher‐order operations, such as executive function and social behaviour (Bolkan et al., 2017; Parnaudeau et al., 2013; Pergola et al., 2018; Rikhye et al., 2018a). The symptomatology of FXS displays a high degree of overlap with functions controlled by thalamocortical circuitry (Holsen et al., 2008; Schmitt et al., 2022), providing strong implications of prefrontal circuit pathology in FXS. Research has revealed disruptions to prefrontal circuitry through changes in both intrinsic and synaptic properties, in addition to mPFC‐mediated learning (Kalmbach et al., 2015; Krueger et al., 2011; Siegel et al., 2017). The importance of MD in executive and affective functions led to our hypothesis that disruptions in MD function might contribute to pathology associated with FXS. We found that *Fmr1* KO MD‐L neurons that project to the mPFC displayed reduced HCN activity compared with WT MD‐L neurons. Further investigation revealed HCN‐channel‐dependent differences in membrane voltage dynamics after the offset of depolarizing and hyperpolarizing current injections manifesting as decreased AHP amplitude and delayed RST.

We identified differences in events following the offset of both hyperpolarizing (RST) and depolarizing (AHP) current stimuli when comparing *Fmr1* KO with WT MD‐L neurons. The AHP amplitude was decreased in *Fmr1* KO MD neurons by ∼20%. The RST was delayed in bursts from *Fmr1* KO neurons by ∼30% when compared with WT control neurons. In a subset of cells, TTX was applied to isolate LTS. We found that RST of calcium spikes was delayed in *Fmr1* KO neurons by ∼35%. These findings suggest that *Fmr1* KO MD‐L neurons might have difficulty in maintaining phase‐locked rhythmic firing, which is crucial for normal thalamocortical circuit function. The thalamus is important for the generation and maintenance of rhythmic activity in the brain (Llinás & Steriade, 2006). Furthermore, rhythmic activity in MD is an integral component to normal higher‐order brain function, particularly working memory (Parnaudeau et al., 2017; Rikhye et al., 2018b) and normal sleep function (Halgren et al., 2019; Mak‐McCully et al., 2017).

Because *Fmr1* KO MD‐L neurons had increased input resistance, we predicted that modelled synaptic currents would show greater summation in *Fmr1* KO compared with WT neurons. However, injected αEPSPs revealed no difference in summation properties at any recorded frequency. This contrasts with observed effects of HCN channel activity on EPSP summation in hippocampal pyramidal neurons (Poolos et al., 2002). It remains unknown how HCN channels localize within the membrane of MD neurons. Dendritic HCN channel localization might impact the differences in αEPSPs observed in somatic recordings (Johnston & Wu, 1994). Although this study focuses on differences in HCN channel activity, it is likely that other channelopathies are present in MD neurons of *Fmr1* KO animals (Brager & Johnston, 2014). For instance, the lack of difference in αEPSP summation between *Fmr1* KO neurons could be explained by increased resting K^+^ conductance in *Fmr1* KO MD‐L neurons. The MD neurons express two‐pore K^+^ (K_2P_) channels, which contribute to the control over state‐dependent firing within thalamic neurons (Bista et al., 2015). Increased activity of these channels could account for the lack of difference in αEPSP summation between WT and *Fmr1* KO MD‐L neurons despite the difference in input resistance reported here.

Recordings from human MD reveal the presence of both sleep spindles and ripples, establishing a role for MD in normal sleep function (Szalárdy et al., 2024). Spindle activity in thalamic neurons is thought to stem from rebound bursting in response to hyperpolarizing GABAergic inputs (Steriade, 2006). Reduced HCN activity in *Fmr1* KO MD‐L neurons contributes to delayed spike timing and reduced AHP amplitude, changes that will functionally impair the ability of a neuron to generate rhythmic bursting activity. Rhythmic bursting in thalamic neurons depends on HCN channel activity, as is shown by disrupted burst generation in an *Hcn4* conditional KO mouse (Zobeiri et al., 2019). Sleep is disrupted in patients with FXS (Budimirovic et al., 2022; Kronk et al., 2010). *Fmr1* KO mice have reduced sleep spindle density and sleep spindle discoordination in cortical areas, including the prefrontal network (Saré et al., 2017). Our results predict shifts in the resonant frequency of MD‐L neurons in *Fmr1* KO mice to lower frequencies. Future work will include explicit measurements of membrane resonance through the injection of oscillating currents at different frequencies (Hu et al., 2002). Our results also predict disruption of rhythmic bursting activity in MD neurons in *Fmr1* KO mice. Future research that is beyond the scope of the present report will be needed to test the relationship between *ex vivo* findings and in vivo circuit‐level activity and behaviour. Activation of G‐protein receptor‐coupled inwardly rectifying K^+^ (GIRK) channels (another contributor to membrane resonance) restored normal cortical sleep spindle activity in *Fmr1* KO mice (Martinez et al., 2024). Our findings suggest enhancing HCN channel activity in MD as a potential therapeutic target for normalizing spindle activity also.

## 5 CONCLUSION

In this study, we performed whole‐cell current‐clamp recordings from CTB‐labelled MD neurons that project to mPFC in WT and *Fmr1* KO mice. The MD‐L→mPFC neurons showed decreased HCN channel activity, whereas MD‐M→mPFC neurons did not. The potential reasons for this are myriad, but given the differences in HCN channel activity between subregions (Lyuboslavsky et al., 2024), we hypothesize that it is related to differences in HCN channel subunit expression between subregions that might be differentially regulated by FMRP. Future work can delineate how these populations of retrogradely labelled neurons correspond to populations defined based on other properties, such as gene expression (Onishi et al., 2021; Schulmann et al., 2024). This study focused on identifying the effects of *Fmr1* KO on intrinsic neuronal properties in MD; however, synaptic dysfunction is widely reported in FXS (Bagni & Zukin, 2019). HCN plays a prominent role in the integration, filtering and coordination of synaptic inputs in neuronal dendrites (Berger et al., 2003; Magee & Cook, 2000; Narayanan & Johnston, 2007; Poolos et al., 2002). Although somatically generated αEPSPs did not reveal differences in subthreshold summation between WT and *Fmr1* KO MD‐L neurons, the localization and dendritic effects of HCN channel expression remain unexplored. Thalamic neurons, through their unique ion channel expression profile, generate and maintain rhythmic activity. Coupled with the widespread interconnectivity between cortical and subcortical brain structures, MD is a promising target for therapeutic intervention for the treatment of symptoms associated with FXS and other neurodevelopmental disorders.

## AUTHOR CONTRIBUTIONS

Gregory J. Ordemann wrote the paper and interpreted and analysed data. Polina Lyuboslavsky performed electrophysiological recordings. Alena Kizimenko performed imaging and projection tracing. Audrey C. Brumback conceived the project, designed all experiments, wrote all code, analysed data and wrote portions of the paper. All authors approved the final version of the manuscript and agree to be accountable for all aspects of the work in ensuring that questions related to the accuracy or integrity of any part of the work are appropriately investigated and resolved. All persons designated as authors qualify for authorship, and all those who qualify for authorship are listed.

## CONFLICT OF INTEREST

The authors have no potential conflicts of interest.

## Data Availability

Source data are available through GitHub.
